# SP1‐mediated lncRNA PVT1 modulates the proliferation and apoptosis of lens epithelial cells in diabetic cataract via miR‐214‐3p/MMP2 axis

**DOI:** 10.1111/jcmm.14762

**Published:** 2019-11-21

**Authors:** Jun Yang, Shaozhen Zhao, Fang Tian

**Affiliations:** ^1^ Tianjin Medical University Eye Hospital Tianjin Medical University Eye Institute & Tianjin Medical University School of Optometry and Ophthalmology Tianjin China

**Keywords:** diabetic cataract, HLE B‐3 cells, human lens epithelial cells, PVT1, SP1

## Abstract

Emerging evidence illustrates the critical roles of long non‐coding RNAs (lncRNAs) in the diabetes. However, the deepgoing regulation of lncRNA PVT1 in the diabetic cataract (DC) is still unclear. Here, present research investigates the pathologic roles and underlying mechanism by which lncRNA PVT1 regulates the DC pathogenesis. Human lens epithelial (HLE) B‐3 cells were induced by the high glucose (HG) to simulate the DC microenvironment models. Results revealed that lncRNA PVT1 expression was up‐regulated in the HG‐induced HLE B‐3 cells as compared to the normal glucose group. Transcription factor SP1 could bind with the promoter region of PVT1 and activate its transcription. Functionally, PVT1 knock‐down could repress the proliferation and promote the apoptosis of HLE B‐3 cells. Mechanistically, PVT1 acted as the ‘miRNA sponge’ to target miR‐214‐3p/MMP2 axis. This finding revealed a novel insight of lncRNA PVT1 for the DC pathogenesis, providing an inspiration for the DC mechanism.

## 1 INTRODUCTION

Diabetes mellitus could arouse a series of complications, including abnormal blood distribution, microcirculation abnormity and metabolic disorders.^1^, ^2^ For the eyes, the terminal circulation anomaly could trigger the cataract. The high glucose (HG) could cause the lens metabolism disorder, thereby inducing the diabetic cataract (DC).^3^, ^4^ Human lens epithelial cells (HLECs) suffer the apoptosis and injure from HG environment in the pathogenesis of DC.^5^, ^6^

Long non‐coding RNAs (lncRNAs) are group of non‐coding transcripts without protein encoding potential, as well as the micro‐RNA (miRNA), circular RNA, piwi‐interacting (piRNA).^7^, ^8^ The increasing roles of lncRNAs in the multisystem disease caused by the diabetes mellitus have been recognized.^9^, ^10^ For example, lncRNA TUG1 regulates the extracellular matrix accumulation, including PAI‐1, TGF‐β1, fibronectin (FN) and collagen IV (Col IV), via mediating micro‐RNA‐377 targeting of PPARγ in diabetic nephropathy.^11^ LncRNA SNHG7 was decreased in human retinal endothelial cells under high glucose stimuli, and SNHG7 overexpression inhibits the high glucose‐induced cell proliferation, migration and angiogenesis by targeting vascular endothelial growth factor (VEGF).^12^ Therefore, lncRNA could wildly regulate the high glucose‐stimulated organ lesions.

The roles of lncRNA PVT1 in the diabetic complication have been illustrated in previous literature. For example, PVT1‐mediated autophagy might ameliorate cognitive impairment and avoid hippocampal neurons impairment by synaptic plasticity and apoptosis in diabetes.^13^ Transcription factor specificity protein 1 (Sp1) is found to participate in the oxidative stress‐driven aberrant Sumoylation signalling of lens epithelial cells.^14^ In the present research, we found that lncRNA PVT1 was significantly up‐regulated in the diabetic cataract tissue and the high glucose‐induced lens epithelial cell line (HLE B3). SP1‐mediated lncRNA PVT1 overexpression modulated the proliferation and apoptosis of HLE B3 cells via miR‐214‐3p/MMP2 axis. This finding revealed a novel insight of lncRNA PVT1 for the DC pathogenesis, providing an inspiration for the DC mechanism.

## 2 MATERIALS AND METHODS

### 2.1 Tissue samples collection

In the initial stage, anterior lens capsule tissues from diabetic cataract patients and normal patient individuals without diabetes mellitus were collected. All these procedures were performed according to the Ethics Committee of Tianjin Medical University Eye Hospital. Each participator had signed the informed consent before the surgery. This study had been approved by the Ethics Committee.

### 2.2 Cells and culture

Human LEC line (HLE B‐3) was provided by the ATCC (CRL‐11421) and cultured in the DMEM medium supplemented with 10% FBS (foetal bovine serum) and 15 mmol/L HEPES as previously described.^15^ HLE B‐3 cell was maintained in an incubator at 37°C with 5% CO_2_.

### 2.3 Transfection

The short hairpin RNA (shRNA), overexpression plasmids (pcDNA3.1 vector) and blank control targeting PVT1 were synthesized by the GENEWIZ. miR‐214‐3p mimics and inhibitors were synthesized by the RiboBio. Cell transfection was conducted using Lipofectamine 2000 (Invitrogen) following the manufacturer's specifications.

### 2.4 RNA isolation and RT‐PCR

Total RNA was extracted from cells using a kit from Qiagen. The extracted RNA (1 μg) was subjected to reversely transcribed using the cDNA using the PrimeScript RT Reagent (TAKARA), and the qPCR was carried out using the SYBR Premix Ex Taq™ (Takara). For the miRNA, qPCR was carried out using Hairpin‐it™ MicroRNAs Quantitation PCR Kit (GenePharma). GAPDH was used as an internal control for lncRNA or mRNA, and U6 was used as the internal control for miRNA. The relative level of RNA was calculated using the 2^−ΔΔCt^ method. The primer sequences were shown in Additional Table S1.

### 2.5 Western blot analysis

Protein was extracted by RIPA lysis buffer with 1% protease inhibitor (Solarbio) from cell lines and tissue. Protein was separated by 10% sodium dodecylsulphate‐polyacrylamide gel electrophoresis (SDS‐PAGE) and then transferred to PVDF member (polyvinylidene difluoride, Millipore). PVDF member was blocked and then incubated with primary antibody (anti‐SP1, Abcam, ab13370, anti‐MMP2, ab37150, 1:1000) overnight at 4°C. Then, the blots were incubated with the appropriate secondary antibodies. The signals were visualized using enhanced chemiluminescence (Thermo Scientific) and recorded on a Gel Doc 2000 imaging scanner (Bio‐Rad).

### 2.6 Cell counting Kit‐8 assay

Cellular proliferation was performed using Cell Counting Kit‐8 (Beyotime) according to instructions. In brief, HKE B3 cells were transfected with corresponding shRNA and seeded into 96‐well plates (1 × 10^3^ cells/well). At 0, 24, 48 and 72 hours after transfection, the vitality was measured with the 10 μL of CCK‐8 (5 mg/mL) and the absorbance was detected using an microplate reader (Bio‐Rad) at a wavelength of 450 nm. Experiment was repeated for three times.

### 2.7 Ethynyl deoxyuridine (Edu) analysis

EdU assay was performed to determine the cell proliferative ability using an EdU Apollo DNA in vitro kit (Ribobio) by the manufacturer's instructions. In brief, HLE B3 cells were, respectively, transfected with corresponding shRNA or controls. After 48 hours after transfection, cells were incubated with EdU (100 μL in 50 μmol/L) per well for 2 hours at 37°C and incubated with glycine (50 μL of 2 mg/mL). Cells were fixed with 4% paraformaldehyde and then treated with 0.5% Triton X‐100 at room temperature. Cells were washed with PBS and stained with anti‐EdU and then incubated with Hoechst 33 342 (100 μL) at room temperature for 30 min. The images were observed under a fluorescent microscope, and the EdU‐positive cells were calculated.

### 2.8 Apoptosis analysis

Apoptosis analysis of HLE B3 cells was determined by flow cytometry. Cells were transfected with shRNAs and then washed with PBS to incubate with Annexin‐V and propidium iodide (Becton Dickinson) according to the manufacturer's instructions. Cells were incubated with the agents for 15 min at room temperature in the dark, and the apoptosis was analysed with a flow cytometric analyses (Attune, Life Technologies). The apoptotic cells were calculated, including early apoptotic cells and advanced apoptotic cells. Experiment was performed in triplicate.

### 2.9 Fluorescence in situ hybridization (FISH)

The FISH was performed as previously described.^16^ The probes targeting the PVT1 (CY3‐labelled PVT1) and miR‐214‐3p (FAM‐labelled miR‐214‐3p) were designed by the GenePharma. In brief, 1 × 10^7^ cells were resuspended in 1 mL ice‐cold RNase‐free PBS, 1 mL buffer and 3 mL RNase‐free water. The probe signals were determined with the Fluorescent in Situ Hybridization Kit (GenePharma) according to the manufacturer's guideline. The cells were visualized under a confocal microscope (Zeiss). The sequences for probes are presented in Table S1.

### 2.10 Luciferase reporter assay

The putative binding sites for SP1 containing PVT1 promoter regions and the potential miR‐214‐3p binding sites or mutant of PVT1 or MMP2 3′‐UTR were cloned into pGL3 plasmid. The desired clone was sequenced and named pGL3‐PVT1‐wild‐type, pGL3‐PVT1‐mutant, pGL3‐MMP2‐wild‐type and pGL3‐MMP2‐mutant. 293T cells were transfected with luciferase reporter plasmid or empty vector with Lipofectamine 2000. Cells were placed on a 24‐well plate and grew till 80% confluence. Forty‐eight hours later, the relative luciferase activities were measured through using Dual‐luciferase Assay System (Promega).

### 2.11 ChIP assay

ChIP assay was carried out using the EZ ChIP™ Chromatin Immunoprecipitation Kit for cell line samples (Millipore) according to the manufacturer's instruction. In brief, the cross‐linked chromatin DNA was sonicated to be fragments (200‐500 bp length). The fragments were immunoprecipitated using the primary antibody (anti‐SP1, Abcam, ChIP Grade, ab13370, 1:1000) and normal IgG as the negative control. Lastly, the immunoprecipitated DNA was quantitatively analysed using the qPCR with SYBR Green Mix (Takara). The primer sequences for the ChIP‐PCR were shown in the Table S1.

### 2.12 Statistical analysis

The statistical analysis was calculated using SPSS version 19.0 software and graphed using the GraphPad Prism 5 software (GraphPad Software Inc). The experimental results were presented as means ± SD and computed using Student's *t* test or one‐way ANOVA. *P*‐values <.05 were considered statistically significant.

## 3 RESULTS

### 3.1 SP1 and lncRNA PVT1 were up‐regulated in the high glucose‐administrated HLE B‐3

**Figure 1:**
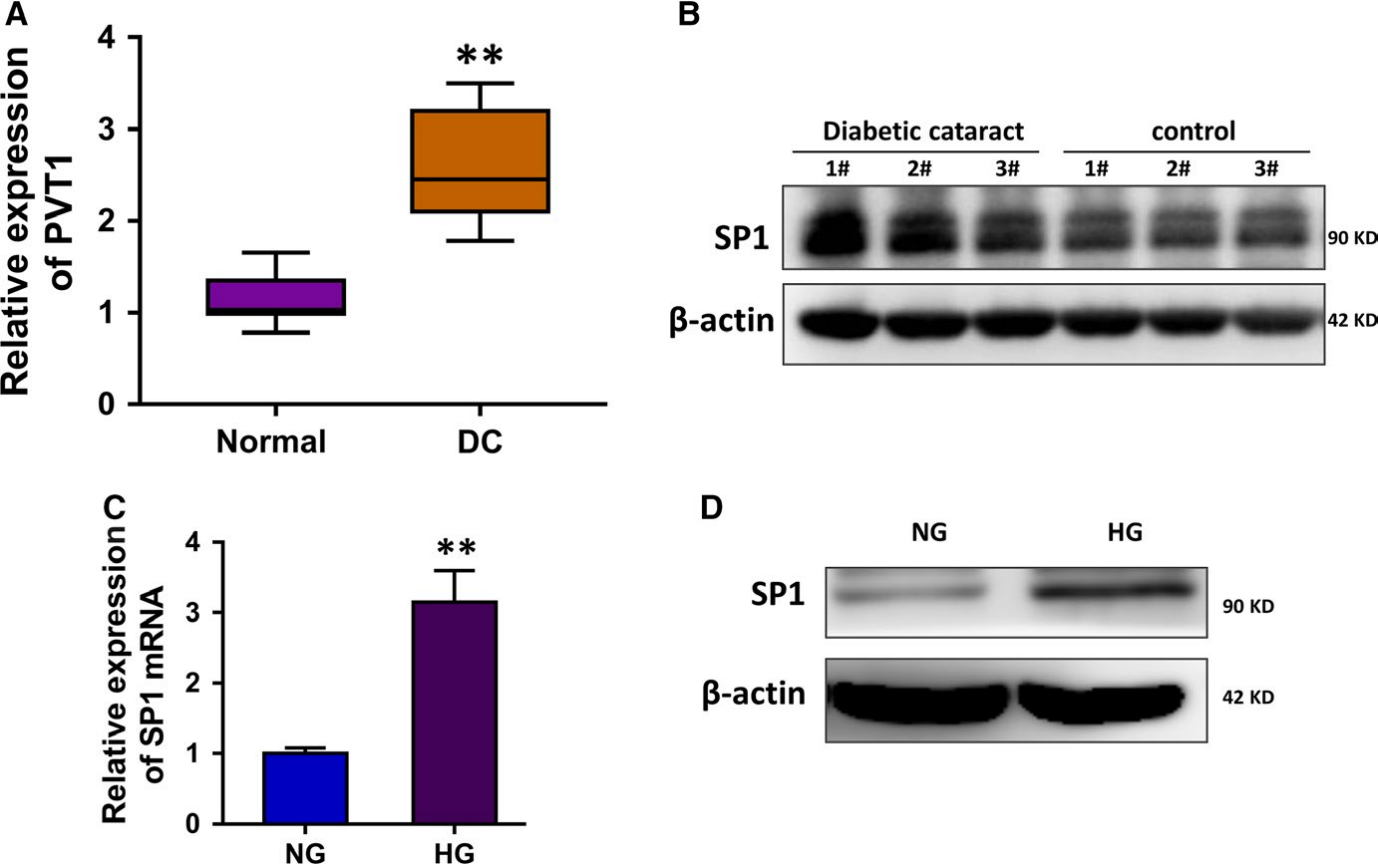
SP1 and lncRNA PVT1 are up‐regulated in the high glucose‐administrated HLE B‐3. A, RT‐PCR revealed the expression levels of lncRNA PVT1 in the DC tissue and normal lens anterior capsule samples. B, Western blot analysis illustrated the SP1 protein in the DC tissue and normal tissue. C, RT‐PCR showed the SP1 mRNA in the HLE B‐3 cells administrated with normal glucose (NG) and high glucose (HG). D, Western blot analysis illustrated the SP1 protein in the HLE B‐3 cells administrated with normal glucose (NG) and high glucose (HG). Data were presented as mean ± SD. **P < .01

In the enrolled diabetic cataract (DC) tissue samples and lens anterior capsule samples, RT‐PCR revealed that the expression levels of lncRNA PVT1 were up‐regulated in the DC (Figure 1A). Western blot analysis found that SP1 protein was up‐regulated in the DC tissue compared to the normal tissue (Figure 1B). The HLE B‐3 cells were administrated with normal glucose (NG) and high glucose (HG) to simulate the diabetic cataract pathological microenvironment (Figure 1C). In the HLE B‐3 cells that administrated with HG, SP1 protein was up‐regulated as compared to the NG administration (Figure 1D). In conclusion, we found that SP1 and lncRNA PVT1 were up‐regulated in the high glucose‐administrated HLE B‐3 cells.

### 3.2 Transcription factor SP1 activated the transcription level of PVT1

**Figure 2:**
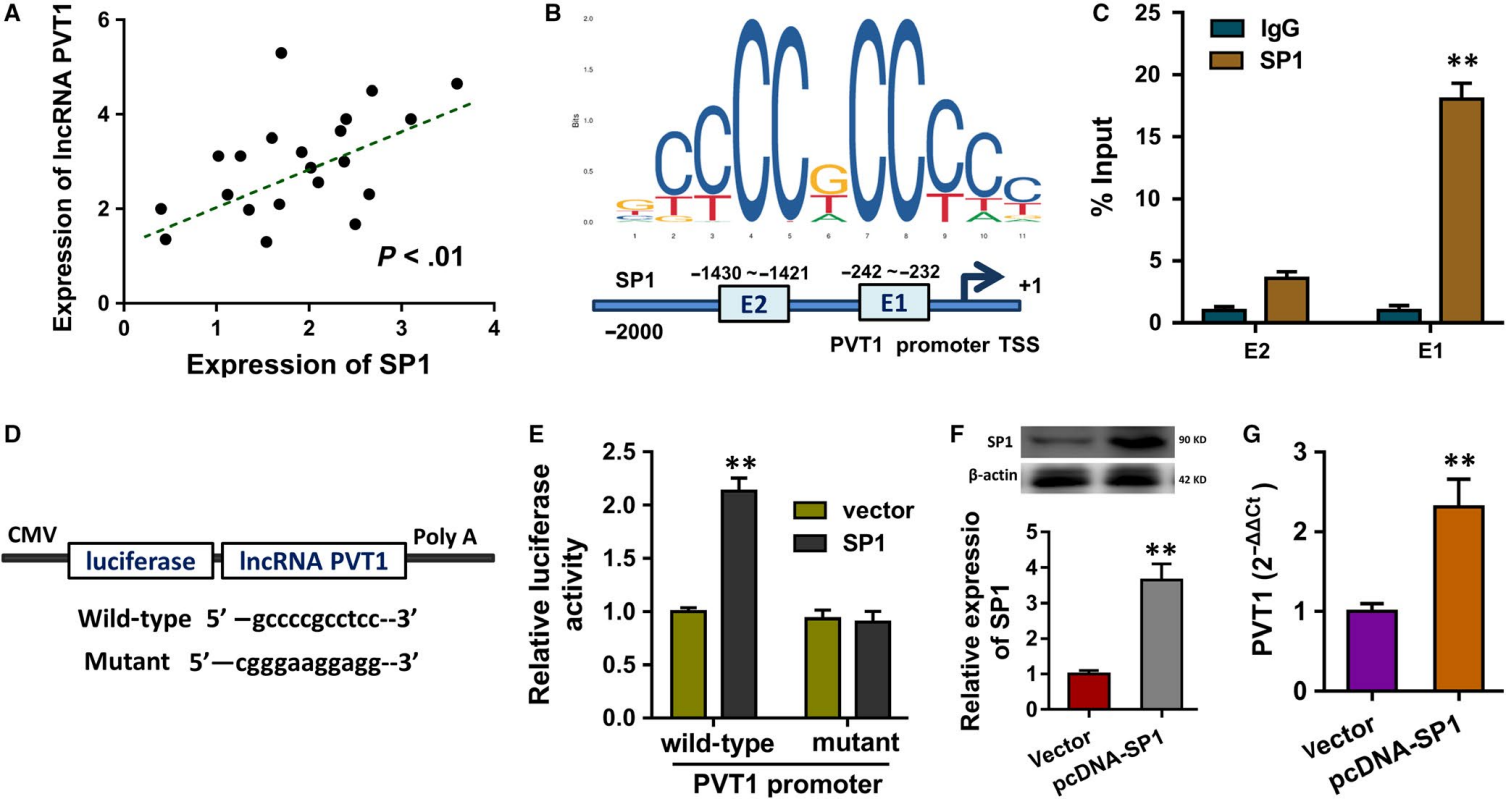
Transcription factor SP1 activated the transcription level of PVT1. A, The correlation analysis of SP1 and lncRNA PVT1 in DC samples was calculated using Spearman's rank methods. B, The JASPAR online tools (http://jaspar.genereg.net/) predicted the possible binding motifs on the promoter region of PVT1 towards transcription factor SP1. C, Chromatin immunoprecipitation (ChIP) assay showed the integration of element with binding motif. D, Luciferase reporter vectors were constructed, including the mutant type and wild‐type. E, The luciferase assay illustrated the activity of wild‐type and SP1 antibody co‐transfection or other control. F, Western blot illustrated the SP1 protein with SP1 overexpression plasmid transfection or not. Data were presented as mean ± SD. **P < .01

In the enrolled tissue samples, the correlation analysis of SP1 and lncRNA PVT1 was calculated using Spearman's rank methods (Figure 2A). The JASPAR online tools (http://jaspar.genereg.net/) predicted that there were two possible binding motifs on the promoter region of PVT1 towards transcription factor SP1 (Figure 2B). Chromatin immunoprecipitation (ChIP) assay showed that SP1 antibody could target the first element of the binding motif (Figure 2C). Luciferase reporter vectors were constructed, including the mutant type and wild‐type, by inserting the sequences containing the first binding motif (Figure 2D). The luciferase assay illustrated that the activity of wild‐type and SP1 antibody co‐transfection was increased, suggesting the molecular binding of SP1 towards PVT1 promoter (Figure 2E). The SP1 overexpression plasmid transfection remarkedly up‐regulated the SP1 protein (Figure 2F). The SP1 overexpression transfection could activate the lncRNA PVT1 level (Figure 2G). In summary, transcription factor SP1 activated the transcription level of PVT1.

### 3.3 PVT1 promoted the apoptosis and repressed the proliferation of HLE B‐3 cells in HG

**Figure 3:**
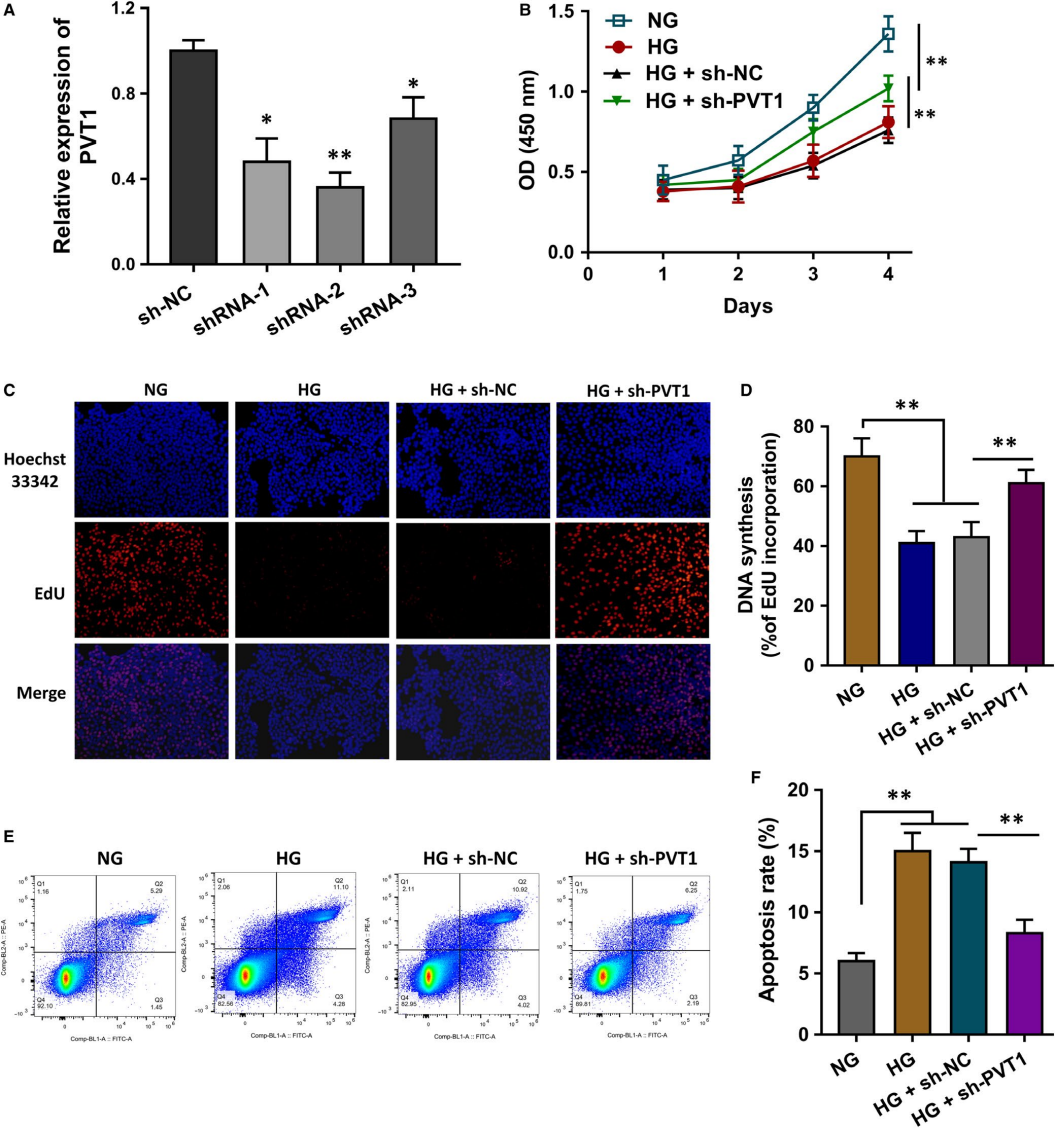
PVT1 promoted the apoptosis and repressed the proliferation of HLE B‐3 cells in HG. A, The short hairpin RNAs (shRNAs) targeting the PVT1 were chemically synthesized to silence its expression. B, CCK‐8 proliferative ability analysis showed the proliferation of HLE B‐3 cells treated with normal glucose (NG), high glucose (HG) and PVT1 silencing transfection. C, D, EdU assay illustrated the proliferation of HLE B‐3 cells treated with normal glucose (NG), high glucose (HG) and PVT1 silencing transfection. E, F, Flow cytometry analysis unveiled the apoptotic rate of HLE B‐3 cells treated with normal glucose (NG), high glucose (HG) and PVT1 silencing transfection. Data were presented as mean ± SD. *P < .1, **P < .01

To investigate the biological roles of lncRNA PVT1 in the HLE B‐3 cells, the short hairpin RNAs (shRNAs) targeting the PVT1 were chemically synthesized to silence its expression (Figure 3A). CCK‐8 proliferative ability analysis showed that the high glucose (HG) treatment triggered the proliferative inhibition of HLE B‐3 cells, and the PVT1 silencing transfection partially recovered the proliferative inhibition (Figure 3B). EdU assay illustrated that the HG induced the proliferation repression of HLE B‐3 cells and the PVT1 silencing transfection promoted the proliferation (Figure 3C, 3D). Flow cytometry analysis for the apoptosis unveiled that the apoptotic rate of HLE B‐3 cells was increased by the HG administration, and the PVT1 silencing transfection reduced the apoptosis (Figure 3E, 3F). In conclusion, lncRNA PVT1 might promote the apoptosis and repress the proliferation of HLE B‐3 cells in HG.

### 3.4 LncRNA PVT1 acted as the sponge of miR‐214‐3p

**Figure 4:**
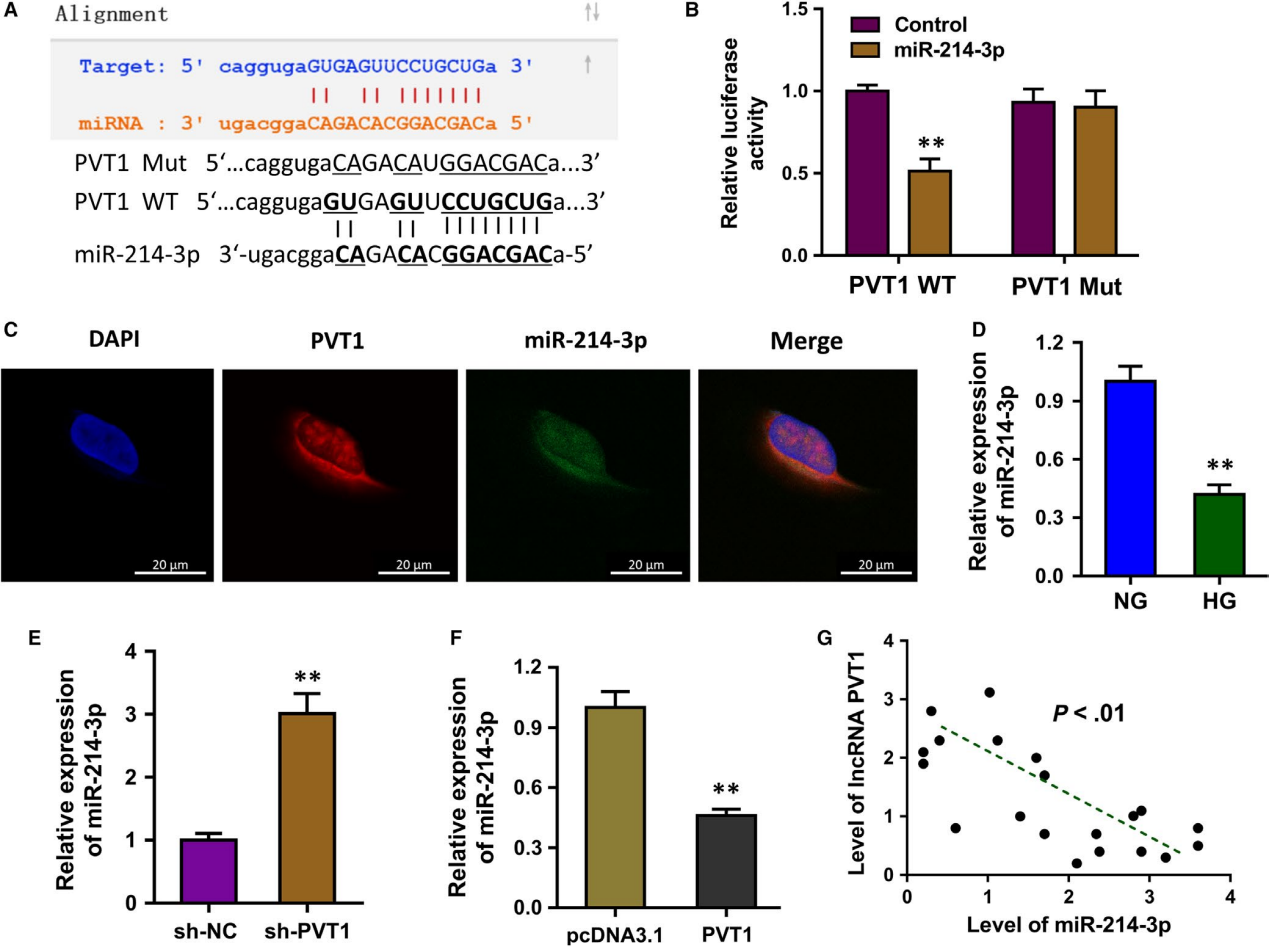
lncRNA PVT1 acted as the sponge of miR‐214‐3p. A, The bioinformatics tools indicated that miR‐214‐3p might function as the possible miRNA target of PVT1 via the binding sites at 3’‐UTR. B, Luciferase reporter showed the luciferase activities of co‐transfection of PVT1 wild‐type and miR‐214‐3p. C, RNA fluorescence in situ hybridization (RNA‐FISH) assay indicated the subcellular location of miR‐214‐3p and PVT1 in HLE B‐3 cells. D, RT‐PCR showed the level of miR‐214‐3p in the HG administration as comparing to NG. E, The expression of miR‐214‐3p with sh‐PVT1 transfection. F, The expression of miR‐214‐3p with PVT1 overexpression plasmid transfection. G, Spearman's rank methods unveiled the negative correlation within PVT1 and miR‐214‐3p was in the DC samples. Data were presented as mean ± SD. **P < .01

The bioinformatics tools indicated that miR‐214‐3p might function as the possible miRNA target of PVT1 (Figure 4A). Luciferase reporter showed that the luciferase activities of co‐transfection of PVT1 wild‐type and miR‐214‐3p were decreased, suggesting the molecular binding within miR‐214‐3p and PVT1 3’‐UTR (Figure 4B). RNA fluorescence in situ hybridization (RNA‐FISH) assay indicated that miR‐214‐3p and PVT1 were both prominently located in the cytoplasm of HLE B‐3 cells (Figure 4C). RT‐PCR showed that the level of miR‐214‐3p was reduced in the HG administration as comparing to NG (Figure 4D). Moreover, the expression of miR‐214‐3p was up‐regulated in the sh‐PVT1 transfection (Figure 4E) and down‐regulated in the PVT1 overexpression plasmid transfection (Figure 4F). Spearman's rank methods unveiled that the expression of PVT1 and miR‐214‐3p was negatively correlated in the DC samples (Figure 4G). Therefore, this finding suggested that lncRNA PVT1 acted as the sponge of miR‐214‐3p.

### 3.5 MMP2 acted as the target of miR‐214‐3p

**Figure 5:**
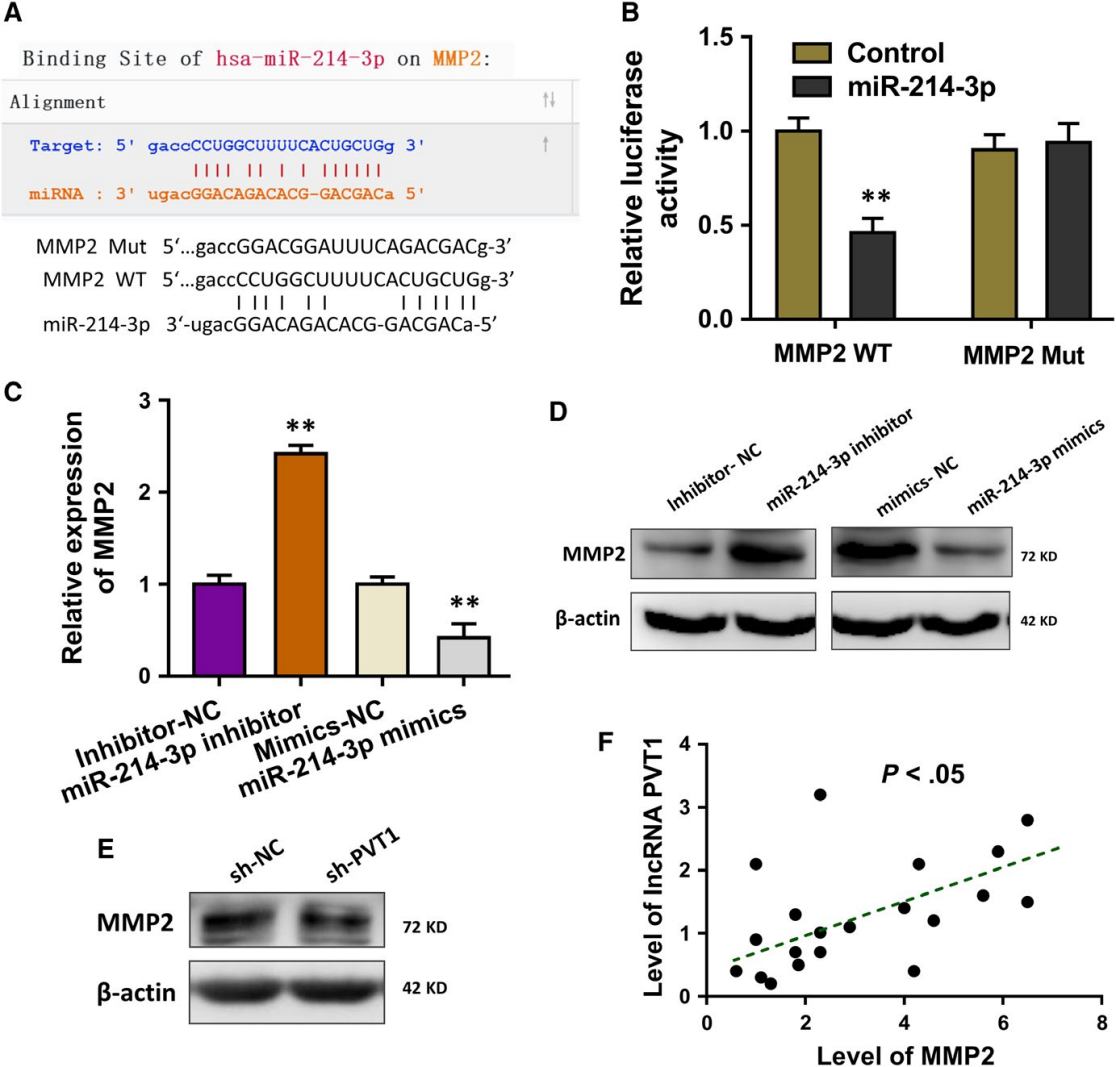
MMP2 acted as the target of miR‐214‐3p. A, The potential target of miR‐214‐3p was predicted using the bioinformatics tools. B, Luciferase reporter gene reporter assay illustrated the interaction within MMP2 and miR‐214‐3p. WT presents the wild‐type plasmids, and the Mut presents the mutant plasmids. C, RT‐qPCR unveiled the MMP2 mRNA level in HLE B‐3 cells transfected with miR‐214‐3p inhibitor or miR‐214‐3p mimics and their controls. D, Western blot analysis indicated the MMP2 protein level in HLE B‐3 cells transfected with miR‐214‐3p inhibitor or miR‐214‐3p mimics and their controls. E, Western blot analysis showed the MMP2 protein levels with PVT1 silencing shRNA transfection. Data were presented as mean ± SD. **P < .01

The potential target of miR‐214‐3p was predicted using the bioinformatics tools and results indicated that MMP2 might act as the target of miR‐214‐3p (Figure 5A). Luciferase reporter gene reporter assay illustrated that MMP2 wild‐type plasmids could significantly target with miR‐214‐3p mimics instead of the mutant plasmids (Figure 5B). RT‐qPCR unveiled that miR‐214‐3p inhibitor transfection enforced the MMP2 mRNA level, and the miR‐214‐3p mimics transfection decreased the MMP2 mRNA level (Figure 5C). Western blot analysis was performed to validate the roles of miR‐214‐3p for MMP2 protein (Figure 5D). Moreover, Western blot analysis showed that PVT1 silencing shRNA transfection remarkedly decreased the MMP2 protein levels (Figure 5E). Spearman's rank methods unveiled that the expression of PVT1 and MMP2 was positively correlated in the DC samples (Figure 5F). Overall, these findings conclude that MMP2 acted as the target of miR‐214‐3p.

## 4 DISCUSSION

With the rapid development of next‐generation sequencing technology and bioinformatics, more and more non‐coding RNAs (ncRNAs) are identified to be involved in the human multiple diseases.^17^, ^18^ LncRNA PVT1 has been reported as a diabetes‐related ncRNA transcript.^19^, ^20^, ^21^ In this research, we found that the lncRNA PVT1 was remarkedly up‐regulated in the diabetic cataract (DC) tissue and high glucose (HG)‐administrated HLE B‐3 cells.

The pathological characteristics of DC are concentrated on the apoptosis and oxidative stress of human lens epithelial cells induced by the HG.^22^, ^23^ In this research, we found that the level of lncRNA PVT1 was significantly up‐regulated in the HG‐induced HLE B‐3 cells. Meanwhile, the proliferation of HLE B‐3 cells was inhibited, and the apoptosis of HLE B‐3 cells was increased in the HG‐induced culture. Then, the knock‐down of HLE B‐3 cells could facilitate the proliferative ability and reduce the apoptosis. This result suggests that the knock‐down of PVT1 could implicate the HG‐induced apoptosis and proliferation of HLE B‐3 cells, acting as the risk factor for the DC.

Mechanically, the transcription factor SP1 was up‐regulated in the HG‐induced HLE B‐3 cells, which was positively correlated with the level of lncRNA PVT1. SP1 functioned as an essential transcription factor in the lens epithelial cells and participated the biological regulation of lens cells.^14^, ^24^, ^25^ In the present study, we found that SP1 could target the promoter region of lncRNA PVT1 and accelerate the transcriptional activity. This finding might unveil the upstream of PVT1 in the DC pathogenesis. In human cancer, SP1 always functions as the oncogenic element in the progression. Analogously, in the HG‐induced HEL B3 cells, the SP1 could serve as the initiating factor to trigger the lncRNA PVT1 abundance.

The RNA‐FISH results revealed that the subcellular location of PVT1 was set in the cytoplasm rather than the nuclear. Besides, the bioinformatics analysis illustrated that miR‐214‐3p might function as one of the targets of lncRNA PVT1. The specific probe targeting miR‐214‐3p in RNA‐FISH revealed that PVT1 and miR‐214‐3p were both located in the cytoplasm of HLE B‐3 cells. Furthermore, MMP2 was identified as the downstream target of PVT1 and miR‐214‐3p. Interaction analysis by RT‐PCR and Western blot illustrated that MMP2 protein level was negatively correlated with miR‐214‐3p, which was positively correlated with PVT1. The modulators matrix metalloproteinases (MMPs) family plays critical roles in the TGFβ2‐mediated matrix contraction.^26^, ^27^, ^28^ Besides, MMP2 is closely correlated with the proliferation of lens epithelial cells. Therefore, lncRNA PVT1 could regulate the HLE B‐3 cell biological activity via modulating the MMP2.

In conclusion, this research unveils the roles of lncRNA PVT1 in the HG‐induced HLE B‐3 cells. In the upstream, transcription factor SP1 could activate the transcriptional level of lncRNA PVT1. LncRNA PVT1 functions as the sponge of miR‐214‐3p to modulate the MMP2, constructing the SP1/PVT1/miR‐214‐3p/MMP2 axis. This finding might be an inspiration for the DC mechanism.

## CONFLICT OF INTEREST

All authors declare no conflicts of interest.

## AUTHOR CONTRIBUTIONS

Jun Yang acts as the major performer for the experiments and writer. Shaozhen Zhao and Fang Tian act as the designer and project leader.

## Data Availability

All the data generated in this study are available upon request.
