# Supplementary material for: SP1‐mediated lncRNA PVT1 modulates the proliferation and apoptosis of lens epithelial cells in diabetic cataract via miR‐214‐3p/MMP2 axis
Source: J Cell Mol Med. 2019 Nov 21;24(1):554–61. doi: 10.1111/jcmm.14762 (PMC6933388; doi:10.1111/jcmm.14762)
Supplement: Supplementary file 1 [file JCMM-24-554-s001.docx]

**Supplement Table 1**. Primers sequences for qRT-PCR, shRNA and FISH.

|  | Sequences 5’-3’ |
| --- | --- |
| PVT1 | forward, 5’- CCAGTGGATTTCCTTGCGG -3'  reverse, 5'- CATCTTGAGGGGCATCTTTTTA -3’ |
| SP1 | forward, 5’- TGGCAGCAGTACCAATGGC-3’  reverse, 5’- CCAGGTAGTCCTGTCAGAACTT-3’ |
| MMP2 | forward, 5’-TACAGGATCATTGGCTACACACC-3’  reverse, 5’-GGTCACATCGCTCCAGACT-3’ |
| GAPDH | forward, 5’-GGAGCGAGATCCCTCCAAAAT -3’  reverse, 5’-GGCTGTTGTCATACTTCTCATGG -3’ |
| sh-PVT1-1 | 5’-ACCGTGCCTAAATCTACG-3’ |
| sh-PVT1-2 | 5’-ACGGCTACTCGGTATTAT-3’ |
| sh-PVT1-3 | 5’-GGGGTTACTTTACACCC-3’ |
| miR-214-3p inhibitor | RiboBio |
| miR-214-3p mimics | RiboBio |
| miR-214-3p | RiboBio |
| ChIP-PCR | forward, 5’-GACCCCTCAGGTAACCATG-3’  reverse, 5’-ATCCTCCGGGCCTTTTGAC-3’ |
| miR-214-3p probe | 5’-ACAGAAAG+UGCU+UCCC+UCAAGAG-3’ |
| PVT1 probe | AGGGUA+UGGCACUCC+UCUCGCCC+  AGCCACGCG+UUGGACUA |
